# “That's like therapy”—A qualitative study on socially disadvantaged women's views on the effects of a community-based participatory research project on their health and health behavior

**DOI:** 10.3389/fpubh.2024.1339556

**Published:** 2024-01-18

**Authors:** Verena Kreiml, Alexandra Sauter, Karim Abu-Omar, Sascha Eickmann, Anne Herrmann-Johns

**Affiliations:** ^1^Department of Epidemiology and Preventive Medicine, Medical Sociology, University of Regensburg, Regensburg, Germany; ^2^Department of Sport Science and Sport, Friedrich-Alexander-University Erlangen-Nuremberg, Erlangen, Germany; ^3^School of Medicine and Public Health, University of Newcastle, Newcastle, NSW, Australia

**Keywords:** physical activity, community-based participatory research, qualitative research, low socioeconomic status, women's health, mental health, social health, health behavior

## Abstract

**Background:**

Regular physical activity has positive effects on both physical and mental health. Nevertheless, socially disadvantaged women are often insufficiently physically active. Through needs-based physical activity offers, community-based participatory research (CBPR) projects have the potential to reach these women and increase the effectiveness of physical activity interventions by supporting women's empowerment, health, and health behaviors. This study aimed to examine socially disadvantaged women's views on the effects of long-term participation in *Bewegung als Investition in Gesundheit* (BIG, i.e., movement as an investment in health), a long-standing German CBPR project, on their health and health behavior.

**Methods:**

Semi-structured qualitative interviews were conducted with 30 participating women at five BIG sites across Germany between April and August 2022. The interviews were recorded, transcribed verbatim, and analyzed using framework analysis.

**Results:**

Women reported that participation in BIG classes contributed to their physical, mental, and social health. For many women, the positive effects on their mental and social wellbeing were most important. In addition to increased fitness and improved physical endurance, many participating women were able to expand their social networks, thus receiving further social support, and improve their self-esteem, self-confidence, and self-efficacy. Furthermore, participation in BIG physical activity classes positively influenced the health awareness of many women helping them to improve their activity level and diet over time.

**Conclusion:**

Our results suggest that CBPR projects, such as the BIG project, can increase physical activity among socially disadvantaged groups and contribute to their overall health and wellbeing. CBPR projects could thus be considered a key element of health promotion for this target group. Future interventional research is required to confirm and further explore the effects of CBPR interventions and to examine whether the effects can be replicated in other settings.

## Introduction

Regular physical activity (PA) has positive effects on both physical and mental health (1). It reduces the risk of obesity, cardiovascular diseases (e.g., coronary heart disease, stroke), and type 2 diabetes mellitus and supports their treatment (1–3). It also improves musculoskeletal health and has a preventive effect on the development of some cancers, such as breast and colon cancer (1–6). Furthermore, PA can help protect against depression, anxiety, and other psychiatric disorders (1, 7–10). The WHO recommends that adults should do at least 150 min of moderate-intensity PA or at least 75 min of vigorous-intensity PA throughout the week (11).

Nevertheless, many adults lack adequate levels of PA (12). PA levels vary depending on various factors, such as gender or socioeconomic status (12–15). According to the German Health Update (GEDA 2019/2020-EHIS), a Germany-wide representative survey of the adult population, only 44.8% of women and 51.2% of men in Germany meet the WHO recommendations (16). In particular, socially disadvantaged women show low rates of PA (17–19). These women may be more likely to be unemployed, be single parents, have a low household income, or have low educational levels (18), resulting in several barriers to engaging in PA (20). Barriers include financial problems, a lack of time, physical and mental illness, a lack of self-confidence, or low motivation (21–24). In addition, especially for women with migration backgrounds, typical barriers to PA include communication issues as well as cultural norms and traditional roles of women (22, 24). Limited language skills prevent them from participating in PA offers, as they do not understand the language spoken there. Culturally inappropriate facilities, such as mixed gender classes and male instructors, and the traditional role of women being mainly responsible for childcare and household duties further restrict the opportunity for PA for these women (22, 24). Needs-based PA programs are required to overcome these barriers and provide socially disadvantaged women with easier access to PA (24, 25).

Community participatory research has become a popular strategy to address this gap. A central element of community-based participatory research (CBPR) is the cooperative planning approach to involve members of the community in the process of setting up preventive services with the help of researchers (26, 27). By establishing PA interventions that are tailored to the needs and demands of the participants, CBPR has the potential to increase the effectiveness of those interventions and address health disparities (27). Furthermore, the participatory approach can be understood as part of the empowerment concept. By working collaboratively with researchers to develop and implement interventions, community members can be empowered to use their own resources and abilities to gain control over their way of living, including health-related behaviors (28). However, when using the CBPR approach, associated challenges must also be considered, such as the process of building and maintaining trust between the members of the CBPR project as well as the long-term continuation of participation (29, 30).

Several studies have shown that participatory interventions have positive effects on health, health behavior (such as PA and nutrition), self-efficacy, and perceived social support of the participating target group (31, 32). The German CBPR project *Bewegung als Investition in Gesundheit* (BIG, i.e., movement as an investment in health) investigated the impact of a participatory PA promotion project on socially disadvantaged women. Participation had positive effects on the general wellbeing and PA behavior of the participating women and contributed to their empowerment (33–38). While several studies have reported these short-term effects of participatory interventions (31–37), there is a lack of studies examining the long-term effects of participation in long-standing CBPR projects on the health and health behavior of socially disadvantaged women (39). These long-term effects might only occur years after the projects have been implemented when women have participated for several years (40, 41). To understand the emergence, maintenance, and interactions of long-term effects and to determine the extent to which long-term participation has further effects on women's health and health behaviors (e.g., on their PA and dietary behavior), in-depth insights into the perceptions of participating women are needed. The present study addresses this research gap by using the long-standing BIG project, which has been running since 2005, as a study context (42). Data collected between 2006 and 2013 already showed that, on average, women had been participating in BIG for more than 2 years at that time (38). Using new qualitative data, this study builds on existing evidence on the effects of CBPR projects on the health and wellbeing of socially disadvantaged women and investigates what further effects long-term participation might have on their health and health behaviors. These insights might enable policy makers to implement tailored and needs-based programs for population groups in difficult life situations in the future. They might also be important for practitioners, e.g. doctors, to be able to respond better to the life situations of women and to incorporate the relevance of PA more strongly into communication with their patients.

This was a qualitative study to explore in-depth the perceptions of socially disadvantaged women regarding:

1) The long-term effects of a CBPR project on their health and wellbeing,
2) The extent to which a CBPR project affects their physical PA behavior and
3) Other effects a CBRR project might have on their health-related behaviors.

## Methods

### Study context: the BIG project

The BIG project was initiated in 2005 by the Department of Sport Science and Sport, Friedrich-Alexander-Universität Erlangen-Nuremberg, Germany (FAU). The aim of BIG is to promote PA among socially disadvantaged women characterized by, e.g., having a low household income, having a migration background, being unemployed, relying on welfare aid, and/or being a single mother (33). To achieve this goal, the project takes a participatory approach. During the implementation process, a cooperative planning group was established, including women, researchers, project coordinators, and local experts (e.g., sports club representatives). This group was responsible for all decisions regarding the planning, implementation, and evaluation of the project and its interventions. At the cooperative planning sessions, the women highlighted their interests, where involved in the development and implementation of activities to promote PA among socially disadvantaged women and helped to decide on instruments for evaluating the project (43). As a result a program of low-threshold PA offerings was developed and implemented, ranging from Zumba, Yoga, and Nordic walking to swimming and cycling classes (36). In addition to the PA offers, other social activities such as women's breakfast sessions, cooking classes, or joint day trips at some BIG locations provide another opportunity for social interaction with other participating women. Since 2005, BIG has been transferred to 22 sites in Germany (44). Of these, seven communities were able to successfully implement the project and keep it running until today. Six sites are currently in the initial phase of the project and in nine communities the project has been terminated (e.g., because of a shortage of staff or insufficient financial resources) (42, 44). The participatory approach was also tried to be maintained after implementation at all sites but in different ways. Some communities continue to hold regular planning meetings with all participating women in order to ascertain their wishes and needs, while at other locations the program is adapted to the wishes of the participants in a more informal setting and when necessary. Furthermore, the participating women at the sites listed in this study are actively involved in the evaluation of BIG (45). The present study is part of the follow-up of the BIG project examining the long-term effects of BIG at all sites (45).

### Study design

We chose a qualitative explorative study design underpinned by a narrative approach (46). Therefore, we conducted semi-standardized qualitative interviews with women participating in BIG classes. The narrative approach was considered appropriate to gain an in-depth understanding of women's perceptions and experiences regarding their health and wellbeing and to explore effects on their health behaviors due to the participation in BIG classes.

### Recruitment

Purposeful sampling was used to include women who had participated in PA classes at one of the five active BIG sites for at least 1 year (47). Local project coordinators, trainers, and other BIG stakeholders (e.g., longtime participants and cultural mediators who support the BIG project) at each site were informed about the details of the interview study by phone, e-mail, or personal meetings and subsequently asked to present them to the class participants. The interview study was further advertised by the research team in BIG classes. Women who were interested in taking part in the current interview study provided their contact details for the research team, who contacted them by phone afterward. They were again informed about the procedure and purpose of the interview study and that participation in the study was voluntary and would not affect their participation in BIG. Women who agreed to participate in the study were given the choice of whether to conduct the interview face-to-face, by phone, or via a videoconferencing platform. The interview mode was decided depending on women's preferences and considering the current pandemic situation. When conduction the interviews, it was again pointed out to the women that the answers should reflect their personal opinions and experiences and that these had no influence on their participation in BIG or the continuation of BIG at their location. An incentive of 30 euros was provided.

### Data collection

The interview guide was based on the RE-AIM model of Glasgow et al. (48). The main questions can be found in Table 1. To pilot test the interview guide, two-phase pretesting was carried out. In the first phase, topics for the interview guide were discussed by the above-mentioned BIG stakeholder group. The women were asked to comment on the suggested topics for the interview guide and to sort them according to relevance, as well as to contribute their own suggestions. In a second step, the finished guidelines were tested in a pilot interview after which only a few fellow-up questions were added. The interviews were conducted by two interviewers (VK and AS). Following the narrative approach (46), the narrative stimulus was initiated by very open initial questions to the women and only supplemented by specific follow-up questions from the interviewer when necessary in order to gain a narrative story of the women's life. To facilitate interviews with non-native speakers, seven interviews were accompanied by interpreters. Women could choose whether their family members (*n* = 4, 57%) or a female master's student with Arab ancestry and experience in qualitative research (*n* = 3, 43%) would serve as interpreters. Lay language was used during the interviews, and questions were kept short and simple. Data collection concluded when data saturation was perceived to be reached, i.e., until further data collection was not perceived to provide additional findings to answer the research questions (49, 50).

**Table 1:** Interview guide questions according to the RE-AIM model of Glasgow et al. (48).

**RE-AIM dimensions**	**Interview guide questions**
Reach	•Why did you decide to participate in BIG? • How important is participation in BIG for you in your everyday life? • Are there factors that make it difficult for you to participate in BIG?
Efficacy	•Which BIG offerings have you been involved in so far? • What changes has participation in BIG classes brought for you? • Would you say that participation in BIG was worth it? If so, for what reason? • Due to the COVID-19 pandemic, the BIG classes could not take place for some time. How was that/is that for you? • To what extent has the COVID-19 pandemic affected your health? • Do you feel that you can express your wishes and interests at the PA classes and other BIG events? • How is your relationship with other BIG participants? • To what extent have you been able to make new acquaintances through BIG? • To what extent has your family life changed because of participating in BIG? (e.g., other family members involved in child care)
Maintenance	•Do you plan to continue with BIG or do more sports independently? • What should be maintained about BIG? • What do you think should be improved?

### Data analysis

All interviews were recorded and transcribed verbatim. Framework analysis, which is assigned to content analysis and is a suitable method to map and interpret qualitative research data, was used for data analysis (51). This systematic approach helped to identify commonalities and differences between the various perspectives of the participating women, allowing for a broad and deep understanding of women's experiences and needs. Five consequential and interconnected steps were followed when applying the framework analysis (51–53). First familiarization served to learn about and understand the interview data. For this purpose, the interviews were conducted, transcribed, and read by the first author (VK). In the second step—coding—an inductive strategy was followed. This involved carefully reading through the interviews line by line and assigning codes for all content and themes that might be relevant to answering the research questions. This is also called “open coding” (51). Thus, three interviews were initially coded by the first author (VK). In the third step—developing the analytical framework—the same interviews were coded again by a second coder (AS). Subsequently, the existing codes were discussed, and compared until consensus could be reached. This resulted in a codebook that could be applied to the coding of the remaining interviews. In the fourth step, the analytical framework was used to index all other transcripts supported by the software Atlas.ti^®^ (version 8). In the fifth step—charting and interpreting—codes were grouped into categories and themes, allowing both similarities and differences between the data to be identified and mapped (51, 53). This involved returning to the transcripts repeatedly for context and content. The analytical process was conducted in German, and only the quotations that were used in this paper were translated into English. Translation was double-checked by a native English speaker.

### Ethical aspects of the study

The study was carried out in accordance with the requirements of the Declaration of Helsinki. The ethics committee of the Friedrich-Alexander-University Erlangen-Nuremberg granted approval for this research (approval number: 247_20 B). All participants gave written consent for their participation and further scientific use of the data. All transcripts were pseudonymized, and only deidentified group data are reported.

## Results

### Participants

All 30 interviews were conducted between April 2022 and August 2022. Of these, 13 took place by telephone, nine via the Zoom^®^ video conferencing platform, and eight face-to-face. The mean interview duration was 38 min (range: 18–73 min).

Participating women came from nine different countries of origin (including Turkey, Germany, and Syria) and were on average 48 years old (range: 22–82 years). Most participants were Muslim (*n* = 18, 60%) and had children (*n* = 23, 77%). 58% of women were unemployed (*n* = 17). Most women joining this study had participated in BIG classes for 3–5 years (n = 13, 43%), with some women having participated in BIG for more than 10 years (*n* = 2, 7%). In addition to PA classes, many women attended other social activities offered by BIG, such as women's breakfast sessions and day trips. Further sociodemographic information and characteristics of the participants can be found in Table 2.

**Table 2:** Sociodemographics and characterization of the participating women.

	**Total**	**Community A**	**Community B**	**Community C**	**Community D**	**Community E**
Number of participants	30	7	9	6	6	2
**Age**						
Average age in years	48.2	54.0	44.9	32.8	53.0	74.5
22–40 years	8	–	2	5	1	–
41–60 years	17	6	7	1	3	–
61–82 years	5	1	–	–	2	2
**Country of origin**						
Turkey	11	–	9	–	2	–
Germany	8	3	–	–	3	2
Syria	5	–	–	5	–	–
France	1	1	–	–	–	–
India	1	1	–	–	–	–
Lebanon	1	–	–	1	–	–
Colombia	1	1	–	–	–	–
Former Yugoslavia	1	1	–	–	–	–
Russia	1	–	–	–	1	–
**Duration of stay in Germany**						
≤ 5 years	4	–	–	4	–	–
≤ 10 years	2	–	–	2	–	–
≤ 20 years	2	1	1	–	–	–
>20 years	12	3	6	–	3	–
Since birth	10	3	2	–	3	2
**Religion**						
Islam	18	1	9	6	2	–
Christianity	8	4	–	–	3	1
Hinduism	1	1	–	–	–	–
Non-denominational	3	1	–	–	1	1
**Occupation**						
Yes	13	5	7	–	1	–
None/housewife/pension	17	2	2	6	5	2
**Relying on welfare aid**						
Yes	3	2	1	–	–	–
No	27	5	8	6	6	2
**Marital status**						
Married	21	3	8	5	5	–
Not married/single/widowed	9	4	1	1	1	2
**Children**						
Yes	23	4	8	5	4	2
No	7	3	1	1	2	–
**Duration of participation in BIG classes**						
≤ 1 year	3	–	1	2	–	–
≤ 2 years	6	2	2	2	–	–
≤ 5 years	13	1	4	2	4	2
≤ 10 years	6	2	2	–	2	–
>10 years	2	2	–	–	–	–

### Perceived effects on health

Women reported the effects that participation in BIG classes had on their physical, mental, and social health, as well as on their PA and dietary behavior. These themes are described in more detail in the following and are summarized in Table 3. In addition, the impact of the pandemic on health and health behaviors and its interaction with the effects of long-term participation in BIG will also be discussed since the COVID-19 pandemic had been impacting women's lives for 2 years at the time of the interviews.

**Table 3:** Overview of the main results of the study.

**Superordinate themes**	**Themes**	**Main results**
Perceived effects on health	Perceived effects on physical health	• Improved physical fitness and endurance • Decrease in pain and positive effects on pre-existing illnesses
	Perceived effects on mental health	• BIG classes as time-out from everyday life and time to focus on yourself • Increased self-esteem, self-confidence, and self-efficacy
	Perceived effects on social health	• Expansion of social networks • Experiencing social support and belonging to a group
	Perceived effects on overall health	• Improved overall well-being and health
Perceived effects on health behaviors	Perceived effects on PA behavior	• Increased integration of PA into their daily lives • Long-term maintenance of participation in BIG classes • Motivation of family members to be more physically active
	Perceived effects on dietary behavior	• Increased awareness of positive effects of a healthy diet on one's own health • Eating and cooking healthier • Implementation of healthier eating behaviors in their families

#### Perceived effects on physical health

Many participating women reported that their physical fitness and wellbeing had improved because of regular class participation. Furthermore, their physical endurance had increased, and they had become more agile since they joined BIG classes. Some women also reported that these effects were prominent in their everyday lives where they generally felt fitter and more energized. A couple of women also attributed these effects to their weight loss following participation. These perceived effects on physical health varied depending on whether women had been physically active before attending BIG classes. For women who had been active in the past, the effects were not as strong as for those who were exercising for the first time.

“*So, I notice that I have more physical endurance and then I take that home with me and I have more energy there again*.” (IP01, 69 years, no migration background, Community E)

“*So, with the stretching exercises, I already noticed that I couldn't move that far at the beginning and that it had already improved through continuous training. And yes, otherwise I've always been pretty athletic. So, regarding physical endurance, I have now not noticed much change*.” (IP27, 30 years, no migration background, Community D)

Many women noted a decrease in pain through regular PA (e.g., back or knee pain). Some also said that participation in BIG had positive effects on their pre-existing illnesses. For example, symptoms caused by asthma, rheumatism, osteoarthritis, or other chronic diseases were perceived to improve. In addition, some women reported that they had to see a doctor or a physiotherapist less often since they started to be more regularly physically active.

“*So, it's just that the pain*—*I had back pain for a while*—*it's completely gone. And if I do the [yoga] regularly, then it stays away*.” (IP15, 45 years, no migration background, Community A)

“*I've hardly been to a physiotherapist for years, or maybe, I don't know once a year or once every 2 years, only if I've truly done something. But other than that, it [*= *the need to seek medical help] can definitely be improved with doing sports*.” (IP16, 49 years, no migration background, Community A)

The COVID-19 pandemic seemed to impact on women's health behavior and physical health, as most BIG classes were canceled during this time. Many women reported that the lack of PA caused them to lose fitness and physical endurance again, that they felt fatigued and weak, and gained weight.

“*Well, yes, so because of the pandemic I stayed at home, I didn't do any sports either, and I ate a lot of sweets, that's why I gained weight*.” (IP02, 22 years, with Syrian migration background, Community C)

“*And during the COVID pandemic, if you don't move the way you move [when you participate in BIG], it's been more limited. You feel more exhausted and tired*.” (IP09, 37 years, with Turkish migration background, Community B)

#### Perceived effects on mental health

Participation in BIG classes also seemed to impact on women's mental health. Almost all women reported that taking part in BIG classes meant having a time-out from everyday life, during which they could focus on themselves rather on family, household, or work.

“*[When I'm swimming] I*'*m completely gone. […] I completely switch-off*.” (IP22, 57 years, with Turkish migration background, Community B)

“*And then that's the time for yourself. You ponder and you come round. And you say: Yes, hello, me too, I'm here too. I'm also functioning. Or I am also important*.” (IP24, 55 years, with Turkish migration background, Community D)

Participation in BIG classes also seemed to increase the self-confidence of some women. This was due to several reasons: Physical changes, such as weight loss or muscle building, made them feel more attractive. Women who had not lived in Germany for long also reported that participation and the associated improvement in German language skills made them become more confident when talking to others in public as they left their known environment more often and thus their “comfort zone” to interact with others they hardly knew. In addition, some women with Muslim faith said they had to be more assertive toward their husbands to keep participating in BIG classes, which increased their self-confidence within the family as well.

“*So, I think [I'm] more open, more open. I dare to go to people more. So, I was always open, but it's easier now. Because back then I was in a new country with a new language and you were in such a small microcosm, yes. […] But there you are out of this microcosm, yes*.” (IP17, 43 years, with French migration background, Community A)

“*So, in the beginning, my husband, he said, ‘Well, we only have Sunday. Stay at home.' And so on and so on. And I said: ‘Let me be for myself for a few hours on Sunday. I'm going.' And he noticed, yes oh I don't comply, I'm going anyway. Since then, he doesn't say anything*.” (IP13, 41 years, with Turkish migration background, Community B)

Most women felt proud of themselves as they managed to participate in a PA promotion project and were able to sustain it over a longer period of time (mainly 3–5 years) without resigning. Some women repeatedly participated in different PA offerings, while others attended the same BIG classes for years. Staying physically active and perceiving the positive effects this had on their health also seemed to improve their self-efficacy. In particular, those who learned a new sport (e.g., swimming, cycling) felt proud as they watched themselves gradually learn a skill they had not previously acquired. For many women, the desire to learn how to swim or ride a bike had been unfulfilled for several years and their self-efficacy was strengthened when they succeeded and fulfilled this wish.

“*I was proud that I kept-up cycling and didn't say again after an hour or after a session, ‘Oh no, I can't do it properly.' Instead, it [*= *the skill to ride a bike] got better each time, and you could see the progress*.” (IP21, 57 years, no migration background, Community A)

Online classes that took place during the COVID-19 pandemic seemed to help some women to get through this particularly difficult time by offering them a break from the stressful and somewhat distressing everyday life characterized by lockdowns and social distancing. Although online classes were offered in four out of the five communities during the pandemic, this offer was used in only one community, while women from the other three communities reported not taking advantage of the online classes. They reported not having enough space at home to be physically active and lacking social interaction with other class participants during the exercises.

“*And especially now with COVID, […] your head is about to explode, until you get that burnout* […]” (IP17, 43 years, with French migration background, Community A)

“*And that [online yoga] provided this power for the whole week where I got my strength back and could focus on me and that […] saved me in this time*.” (IP17, 43 years, with French migration background, Community A)

#### Perceived effects on social health

Women reported several impacts BIG had on their social health, i.e., the social experiences in terms of the social support and contacts women were able to have due to their participation in BIG. Many women appreciated the familiar atmosphere within the classes and the social exchange with other class participants or trainers which was often described as “family” involving strong bonds, trust, and reciprocity. They reported feeling comfortable and safe in the classes, which allowed them to talk openly about various topics, including conflicts with their partners or issues related to raising their children. Furthermore, almost all women described that they were able to expand their social networks and some even made friends beyond BIG participation. These social experiences were the unique selling point for many women, which is why they chose to participate in BIG classes year after year instead of attending PA programs offered by other local PA providers.

“*We are just like a family there. We can talk openly about everything*.” (IP09, 37 years, with Turkish migration background, Community B)

Especially women who had not lived in Germany for long as well as women with pre-existing mental illnesses often felt socially isolated and as if they did not belong to any social group before participating in BIG. Participation made it easier for them to meet peers and make contact with others whom they could ask for help related to problems in everyday life, such as arranging doctors' appointments and finding their way around the German healthcare system. By experiencing social support and belonging to a group, women seemed to feel more integrated into society and, through the resulting increased psychological wellbeing, also strengthened in their mental health.

“[…] *even if you need help with paperwork and other things, or with using the Internet. I can ask someone*.” (IP01, 69 years, no migration background, Community E)

“*And I notice that when I come back from the BIG classes, it is good for my soul because I have laughed with others and because I have talked with others*.” (IP04, 59 years, with Indian migration background, Community A)

Following the cancellation of classes during the COVID-19 pandemic, many women felt isolated and lonely, especially because they lacked social contact and exchange within the classes. Despite this, many women remained in contact with other participants during the pandemic through online communication platforms (e.g., WhatsApp), which could not replace face-to-face exchanges but did provide some way to positively influence their social health.

“*First, I missed the physical activity [during the pandemic]. I did not move a lot. Then, the social contacts were also missing. That was the second thing I missed, yes*.” (IP22, 57 years, with Turkish migration background, Community B)

#### Perceived effects on overall health

Women across all BIG sites felt that regular participation in BIG classes over years had positive effects on all three areas of health: physical, mental, and social health. This led to an improvement in overall wellbeing and health.

“*So that's [*= *participation in BIG] just for the body and […] for the soul, as you think. It's good for everything. That's just fun*.” (IP07, 46 years, with Turkish migration background, Community B)

“*Like therapy. This is like a therapy, really. And we can also talk to the others and chat a bit, smile while doing sports too. Everything is possible*.” (IP13, 41 years, with Turkish migration background, Community B)

### Perceived effects on health behaviors

#### Perceived effects on PA behavior

Women from all sites reported that they were able to integrate more PA into their daily lives and leisure time. Women who participated in swimming or cycling classes seemed to be empowered to apply the newly learned skills into other areas of life, for example, by going shopping by bike or swimming on their own. Additionally, some women were able to learn exercises and movement sequences in the classes and perform them independently at home. For other women, the increase in PA in their everyday lives was due to improved awareness of the importance of exercising. This was supported by some trainers explaining the structure and functions of the human body and reminding women to take care of their bodies in everyday life. The rise in awareness of regarding the health benefits of PA led some women to change their routines and, for example, to use their bicycles instead of their cars and to attend additional PA offers outside of BIG (such as Zumba classes).

“*So, I very rarely take the car with my husband, unless we have to go very far. And I go everywhere by bicycle. I didn't do that [before BIG]*.” (IP04, 59 years, with Indian migration background, Community A)

“*Now we have [a trainer] who explains the entire body structure very well and what you should pay attention to. And you can use that very well [to improve your body posture]*.” (IP30, 82 years, no migration background, Community D)

Most women were aware of the positive effects of PA on their health, which motivated them to continue their participation in BIG for years. Many women reported that they did not want to switch to other local PA providers but wanted to stay within the BIG classes. The main reasons for this were the cultural sensitivity of the BIG classes (e.g., only female participants and trainers), low-cost participation in BIG, and the social experiences mentioned above. Many wished to have classes offered more often to build on the health effects they had already experienced through PA.

“*And I wanted only women to be [at the swimming classes]. Additionally, the fee at BIG is very good, it fits perfectly. […] When I found the [BIG] class, I was totally thrilled. I waited for at least a year to find one*.” (IP14, 44 years, with Turkish migration background, Community B)

“*So, I'm happy and enjoy doing it [*= *BIG classes]. And as long as my health allows me to do that, I'll be there […]. As long as the class is offered*.” (IP30, 82 years, no migration background, Community D)

“*Of course, it would be better if you had [BIG classes] more often, at least two or three times a week. Then, you'd be even fitter, maybe*.” (IP12, 52 years, with Turkish migration background, Community B)

Some women also reported that they stayed physically active during the COVID-19 pandemic (e.g., by doing hikes and runs). Nevertheless, most were only able to be physically active at the beginning of the pandemic until they stopped completely. Many women explained that during the pandemic, they no longer had the strength to engage in PA, as their daily lives became more stressful and draining (e.g., due to lack of child care and lack of time for themselves). In addition, it was difficult to motivate themselves to be physically active on their own, as they were accustomed to exercising in groups with other women at regular class times. Few women did not even attempt to bridge the break in BIG classes with independent exercise, for example, due to the closure of swimming pools.

“*We started that during COVID, walking, Mondays, always walking. Because there were no classes*.” (IP04, 59 years, with Indian migration background, Community A)

Participation in BIG not only seemed to influence women's own PA behavior but also appeared to have effects on other family members, positively impacting their family health. Some women managed to motivate their partners to become more physically active, for example, by going for walks or hikes together. Women who had not lived in Germany for long and had young children reported that they could increase the level of PA of the entire family by undertaking trips together, such as bicycle tours.

“*Now I'm more outside with my kids. We go for bike rides. That's also an effect of BIG*.” (IP05, 34 years, with Syrian migration background, Community C)

#### Perceived effects on dietary behavior

For some women, participation in BIG also seemed to affect their dietary behavior. They reported eating more vegetables and fruit while avoiding sweets and fast food. Some women were motivated to eat healthier simply by participating in classes and being more physically active. Women also shared information about healthy nutrition and recipes during and after the classes. At some sites, the trainers also informed women about the benefits of healthy eating in combination with PA and motivated them to do so to achieve even better effects on their health. However, there were also some women who did not notice any changes in their dietary behavior.

“*If you've already done sports, you don't necessarily go for the premade lasagna afterwards, but maybe eat a bit healthier. I think that you become a bit more health conscious overall*.” (IP16, 49 years, no migration background, Community A)

“*And then also nutrition, yes. ‘What did you cook today? And there's so much fat in this and there are so many calories in that. Try it the other way around.' And so on, yeah exactly.”* (IP09, 37 years, with Turkish migration background, Community B)

The changes regarding nutrition seemed to have an impact on women's families as well. Some women were able to implement healthier eating behaviors in their families, so their children also ate fewer sweets, for example. This seemed to be especially successful when their children were very young and simply accepted the new eating habits or when older children had their own health problems they wanted to improve, such as obesity. However, other women reported that their children and partners refused to eat the new types of meals (e.g., due to skepticism or insufficient knowledge about the positive effects of healthy eating), often forcing women to return to former eating habits because it would have been too time-consuming to prepare two different meals.

“*The bad thing is when you cook much healthier or more health consciously that what I cook doesn't suit my family. And for me, it is then double effort to cook for me and for them. And for me, that is associated with so much stress that somehow I say, I don't care what I cook for myself, the main thing is that the family is fed*.” (IP29, 43 years, with Russian migration background, Community D)

## Discussion

### Key findings

In this qualitative study, we examined in-depth the effects of long-term participation (i.e., on average 3–5 years) in PA classes and other social activities that were set up in a participatory process between women, community officials, and researchers as part of a CBPR project on the health, wellbeing, and health behavior of socially disadvantaged women. We conducted and analyzed 30 semi-structured interviews with participating women from five active BIG sites across Germany. The results of our study show that participation in BIG classes contributed to the improvement of women's physical, mental, and social health. For many women, the positive effects on their social and mental wellbeing were most noticeable. Through social support and building a sense of community within the classes, most women were able to expand their social network, which was particularly important for women who felt socially isolated prior to participation (e.g., recently immigrated women, women with pre-existing mental illnesses). Increased self-esteem, self-confidence, and self-efficacy also contributed to improvements in women's mental health. Furthermore, participation in BIG classes positively influenced the health awareness of many women leading them to make changes in their PA and dietary behavior. Some women were also able to support their family members in PA, dietary, and health behaviors, which helped improve family health. Despite different offers of PA classes at each BIG site (e.g., swimming, yoga, cycling), these effects could be observed in women of all five BIG communities, suggesting a successful transferability of the BIG approach from one community to another. Our findings also suggest that the BIG project not only promotes the uptake of PA but also helps improve other areas of health and wellbeing among socially disadvantaged women by providing them with a social environment in which they feel comfortable, supported, and empowered to improve their health and the health of their loved ones.

### Comparison with other studies

The findings of our study align with those of previous studies on the BIG project. For instance, studies by Röger et al. (35) and Rütten et al. (36) showed that involvement in the BIG project results in greater empowerment for women who face social disadvantages. This empowerment manifests as increased self-efficacy and self-confidence (35, 36). Sauter et al. (37) analyzed qualitative interviews conducted at five BIG sites between 2007 and 2011 and confirmed the positive effects on their individual empowerment (e.g., expansion of their social network and increased integration of PA into their daily lives). They also emphasized the impact on women's organizational (e. g., communicating their desires and requirements to others) and community empowerment (e.g., developing advanced problem-solving skills) (37). The transferability of the BIG approach was observed among the examined communities (37). Furthermore, quantitative data collected at two BIG sites between 2006 and 2013 reported improved health status and increased PA among participating women compared to before BIG participation (38). The present study adds to this existing evidence that many of these effects (35–38) were also observed among long-term participants and had positive effects on their physical, mental, and social health. In addition, we identified further effects on the health behavior of participating women, such as changes in PA and dietary behaviors.

Similar effects were found in the Colombian study by Rubio et al. (54). In addition to women's increased levels of PA (55, 56), other effects of participation in community-based PA programs included broadening and strengthening of social ties, increased motivation for PA, awareness of others' wellbeing and improved mental health (54).

A Swedish project aiming to promote PA among socially disadvantaged women (57) also showed that participation in a PA intervention contributed to improvements in the physical, mental, and social health of the participating women. Furthermore, participation empowered them to gain control over their health and wellbeing, so that most of the women remained physically active even during the COVID-19 pandemic (57). This is in contrast to our study, which highlighted that participating women were able to integrate higher levels of PA into their daily lives and leisure time, but only a few maintained these levels during the COVID-19 pandemic when BIG classes had to be canceled. This may be due to differences regarding the project approach but also to the timing of data collection: While Ramji et al. (57) conducted the interviews at the beginning of the pandemic when women in our study also reported still being physically active, our interviews were conducted at the end of the pandemic when many women had already lost motivation to become and remain physically active on their own due to lack of social interactions during PA and the stressful and exhausting time during the pandemic (e.g., due to lack of child care and lack of time for themselves).

A Spanish PA intervention showed that participation improved both PA-related behaviors and dietary behaviors (58). Social support from family members was critical in improving motivation for leisure-time PA and continuing to be physically active after the intervention (59). This is in line with our study, which suggests that receiving social support from partners and children played an important role in the implementation and maintenance of health behaviors such as PA and dietary behavior.

In addition, some women were even able to motivate their partners and children to be physically active (e.g., hiking or biking together) or to adopt the women's healthier eating habits. This led to an improvement in the PA and dietary behavior of family members of participating women. Lo et al. (60) examined the effects that women's participation in a health-promoting CBPR project in the United States had on the PA and dietary behavior of people in their social environment, including family members, friends, and coworkers. However, they could not demonstrate similar effects, probably due to differences in setting and population (e.g., use of a quantitative study design, sedentary women as target population of the CBPR project and examination of effects on adults from across the women's social network) (60). Therefore, further research is required to further investigate the transfer of effects from socially disadvantaged women participating in CBPR projects to their families.

### Strengths and limitations

Interviews were conducted with a heterogeneous group of women from different sociodemographic backgrounds and with an average participation duration of 3–5 years allowing us to obtain a comprehensive picture of the long-term impact of BIG on socially disadvantaged women. Care was taken to conduct the interviews in a culturally sensitive manner. Members of the research team who were in direct contact with participating women were female, which was especially important for women with Muslim faith. In addition, our female interpreter with Arab ancestry seemed to help create a trusting atmosphere during the interviews. For example, some women who were normally veiled in public removed their headscarves during interviews to show that they felt comfortable and protected in the interview situation. Women were allowed to decide on the mode (i.e., phone, video call, face-to-face), place, and timing of the interviews, and the interpreter if necessary. This made it easier for women to integrate the interviews into their daily lives. For example, phone interviews allowed women who did not want to show their faces for religious reasons to participate in the study. Nevertheless, there might be differences in data quality between interviews by phone, video call, or face-to-face. However, after analyzing the data there is no indication that interviews per phone or video call are less valuable than face-to-face interviews (e.g., no significant difference in the length of the interviews and topics discussed). In addition, there is a lack of evidence that interviews by phone provide lower data quality than face-to-face interviews (61, 62).

Limitations of this study may have resulted from family members of study participants who acted as interpreters and only translated the meaning of the conversations, not the verbatim version, which might have resulted in data being lost or slightly changed. To control for this, the research team highlighted that the questions should be translated as literally as possible. Furthermore, member checking was not possible due to time constraints. Another limitation of this study may be that, for reasons of social desirability, women might have talked better about the classes and the effects achieved than they were. The interviewers tried to counteract this phenomenon by repeatedly pointing out that the women were free to express their views and opinions and that they do not have to fear negative consequences if they talk negatively about the BIG project. In addition, the sample in our study consisted mostly of women who successfully participated in BIG for an extended period of time. Thus, the experiences of women who dropped out of BIG participation early could not be considered in this study. Finally, our results are based on the personal and subjective perceptions of participating women and not on objective measurements (e.g., accelerometers). This is in line with the aim of our study to gain in-depth insights into women's personal experiences to achieve a broad and deep understanding of the possible effects of long-term participation in BIG. However, further interventional research is required to further assess and confirm our findings.

### Implication for future research and practice

Our findings suggest that participation in BIG classes positively influenced women's physical, mental, and social health. As a result of the change in health awareness, many women were motivated to attend BIG classes regularly, to remain physically active outside of classes and to pay more attention to healthy eating. Some women were also able to transfer these effects to their families so that the PA and dietary behavior of some partners and children were positively influenced as well. However, some family members rejected the woman's new behaviors and thus forcing them to return to old PA and eating habits. For example, women who were cooking healthy meals had to stop doing so if the new meals were not accepted by other family members. Future research is needed to understand the facilitators and barriers regarding the transfer of effects to family members. Furthermore, involving partners and children in family-based interventions could help make them aware of the importance of PA and other health-related behaviors and motivate them to support the behavioral changes of participating women and to incorporate these behaviors into their lives themselves.

Participating women who received advice from their trainers on how to cook healthier or on how to incorporate specific parts of the body into exercises at home seemed to integrate these health behaviors more sustainably into their daily routines and achieved stronger and longer-lasting effects on their health (e.g., reduction of back pain). This suggests that combining PA classes with information on the human body, nutrition, and other health-related topics could contribute to improved health awareness and behavior among socially disadvantaged women.

Overall, the findings of our study highlight that participation in long-lasting CBPR projects, such as the BIG project, can have a positive and lasting impact on the health and health-related behavior of socially disadvantaged women. The main motivators for long-term participation in BIG were the perceived benefits to their physical, mental, and social health. In addition, the cultural sensitivity of the PA classes and the reduced cost of participation were important facilitators for the initiation of participation in BIG but also contributed to the maintenance of participation over years. Therefore, CBPR projects that are tailored to the needs and demands of the participants could be considered a key element of health promotion for socially disadvantaged groups.

They may even have the potential to be used as an official health promotion tool. For example, participatory interventions could be integrated into the concept of social prescribing. This involves issuing a “social prescription” as a referral to a nonmedical intervention to support people with a range of social, emotional, and practical needs and improve their wellbeing and health behaviors (63, 64). These interventions are typically provided by the voluntary and community sectors and include multiple activities, such as PA (63, 64).

For this to occur, future research should focus on interventional studies further investigating the effects of CBPR interventions to achieve level 1 evidence. Further studies should also assess whether the observed effects can be transferred to other socially disadvantaged groups [e.g., migrant groups who have more recently arrived in Germany, such as Ukrainian refugees (65)].

## Conclusion

In this qualitative study, we explored in-depth the perceptions of socially disadvantaged women regarding the effects of long-term participation in BIG, a long-standing German CBPR project, on their health and health behaviors. Our results suggest that participation in BIG classes improved women's physical, mental, and social health with effects on mental and social wellbeing perceived to be most important by women. Most women were able to expand their social networks, receive further social support by belonging to the “BIG family” and strengthen their self-esteem, self-confidence, and self-efficacy. In addition, many women changed their PA and dietary behaviors due to the increased health awareness they gained from participating in BIG. Some women were also able to communicate these changes in PA and dietary behaviors to their families. Needs-based CBPR projects to promote PA seem to have the potential to contribute holistically and sustainably to health promotion among socially disadvantaged women. Future interventional research should further evaluate the effects of CBPR interventions and examine whether these effects can be replicated in other target groups.

## Data availability statement

The raw data supporting the conclusions of this article will be made available by the authors, without undue reservation.

## Ethics statement

The studies involving humans were approved by Ethics Committee of the Friedrich-Alexander-University Erlangen-Nuremberg. The studies were conducted in accordance with the local legislation and institutional requirements. The participants provided their written informed consent to participate in this study.

## Author contributions

VK: Writing—original draft, Data curation, Formal analysis, Investigation, Project administration, Visualization. AS: Writing—review & editing, Conceptualization, Formal analysis, Investigation, Project administration, Supervision, Funding acquisition. KA-O: Writing—review & editing. SE: Writing—review & editing, Supervision. AH-J: Writing—review & editing, Conceptualization, Supervision.
